# Supplementary material for: The small eye phenotype in the EPIC-Norfolk eye study: prevalence and visual impairment in microphthalmos and nanophthalmos
Source: BMJ Open. 2013 Jul 23;3(7):e003280. doi: 10.1136/bmjopen-2013-003280 (PMC3731707; doi:10.1136/bmjopen-2013-003280)
Supplement: Author's manuscript [file bmjopen-2013-003280.draft_revisions.pdf]

The Small Eye Phenotype in the EPIC-Norfolk Eye Study: Prevalence and Visual Impairment in Microphthalmos and Nanophthalmos.

Journal:	*BMJ Open*
Manuscript ID:	bmjopen-2013-003280
Article Type:	Research
Date Submitted by the Author:	22-May-2013

Complete List of Authors:	Day, Alex; UCL Institute of Ophthalmology,<br>Khawaja, Anthony; University of Cambridge School of Clinical Medicine, Cambridge, United Kingdom, Department of Public Health and Primary Care, Institute of Public Health<br>Peto, Tunde; The NIHR Biomedical Research Centre at Moorfields Eye Hospital NHS Foundation Trust and UCL Institute of Ophthalmology.,<br>Hayat, Shabina; University of Cambridge School of Clinical Medicine, Cambridge, United Kingdom, Department of Public Health and Primary Care, Institute of Public Health<br>Luben, Robert; University of Cambridge School of Clinical Medicine, Cambridge, United Kingdom, Department of Public Health and Primary Care, Institute of Public Health<br>Broadway, David; Norfolk & Norwich University Hospital, Dept. Ophthalmology<br>Khaw, KayTee; University of Cambridge School of Clinical Medicine, Cambridge, United Kingdom, Department of Public Health and Primary Care, Institute of Public Health; University of Cambridge, Clinical Medicine<br>Foster, Paul; Institute of Ophthalmology, Division of of Epidemiology; The NIHR Biomedical Research Centre at Moorfields Eye Hospital NHS Foundation Trust and UCL Institute of Ophthalmology.,

**Primary Subject Heading**:	Ophthalmology
Secondary Subject Heading:	Epidemiology
Keywords:	OPHTHALMOLOGY, EPIDEMIOLOGY, Glaucoma < OPHTHALMOLOGY

SCHOLARONE™
Manuscripts

The Small Eye Phenotype in the EPIC-Norfolk Eye Study: Prevalence and Visual Impairment in Microphthalmos and Nanophthalmos.

AUTHORS

Alexander C Day[1], Anthony P Khawaja[2], Tunde Peto[1], Shabina Hayat[2], Robert Luben[2], David C Broadway[3], Kay-Tee Khaw[2], Paul J Foster[1]

INSTITUTIONS

[1]The NIHR Biomedical Research Centre at Moorfields Eye Hospital NHS Foundation Trust and UCL Institute of Ophthalmology, London, United Kingdom

[2]Department of Public Health and Primary Care, Institute of Public Health, University of Cambridge School of Clinical Medicine, Cambridge, United Kingdom

[3]Department of Ophthalmology, Norfolk and Norwich University Hospital NHS Foundation Trust and University of East Anglia, Norwich, United Kingdom

CORRESPONDENCE

Professor Paul Foster

UCL Institute of Ophthalmology

11-43 Bath Street

London

EC1V 9EL

Tel. 00 44 (0)20 7608 6901

Email: p.foster@ucl.ac.uk

For peer review only

KEYWORDS:

Microphthalmos [MeSH]

Nanophthalmos [MeSH]

Epidemiology [MeSH]

Axial length, eye [MeSH]

Visual acuity [MeSH]

For peer review only

Abstract:

Objective:

To describe the prevalence and phenotypic characteristics of small eyes in the EPIC-Norfolk Eye Study.

Design:

Community cross-sectional study.

Setting:

East England population (Norwich, Norfolk and surrounding area).

Participants:

8033 participants aged 48-91 years old from the EPIC-Norfolk Eye Study, Norfolk, United Kingdom with axial length measurements. Participants underwent a standardized ocular examination including visual acuity (LogMAR), ocular biometry, non-contact tonometry, auto-refraction and fundal photography. A small eye phenotype was defined as a participant with one or both eyes with axial length of <21.00mm.

Outcome measures:

Prevalence of small eyes, proportion with visual impairment, demographic and biometric factors.

Results:

Ninety-six participants (1.20%, 95% CI: 0.98 to 1.46) had an eye with axial length less than 21.00mm, of which 74 (77%) were female. Prevalence values for shorter axial lengths were <20.00mm: 0.27% (0.18 to 0.41); <19.00mm: 0.17% (0.11 to 0.29); <18.00mm: 0.14% (0.08 to 0.25). Two participants (2.1%) had low vision (presenting visual acuity >0.48 LogMAR) and 1 participant was blind (>1.3 logMAR). The prevalence of unilateral visual impairment was higher in participants with a small eye. Multiple logistic regression modeling showed presence of a small eye to be significantly associated with shorter height, lower body mass index, higher systolic blood pressure and lower intraocular pressure.

Conclusions:

The prevalence of people with small eyes is higher than previously thought. Whilst small eyes were more common in women, this appears to be related to shorter height and lower body mass index. Participants with small eyes were more likely to be blind or to have unilateral visual impairment.

Article summary:

Article focus:

- The European Prospective Investigation of Cancer-Norfolk Eye Study is part of a European population-based cohort study, with participants now aged 48-92 years old.
- This paper describes the prevalence of small eyes, proportion with visual impairment, and associated demographic and biometric factors.

Key messages:

- Ninety-six participants out of 8033 (1.20%, 95% CI: 0.98 to 1.46) had an eye with axial length less than 21.00mm, of which 74 (77%) were female.
- People with small eyes appear more likely to be blind or have unilateral visual impairment. Presence of a small eye is associated with shorter height, lower body mass index, higher systolic blood pressure and lower intraocular pressure.
- There are no standardized definitions for microphthalmos or nanophthalmos

Strengths and limitations of this study:

- Large population based study sample.
- The included population sample may have healthy volunteer bias.
- The identified associations are cross-sectional rather than longitudinal.

For peer review only

Introduction:

The small eye phenotype ranges from anophthalmos to nanophthalmos and microphthalmos. The latter two conditions are typically considered to be synonymous[1] and are subdivided into simplex[2] and complex[3] depending on the presence of other associated ocular or systemic abnormalities. There is minimal adult data on the prevalence of this phenotype with estimated birth prevalences for microphthalmos being 0.002 to 0.017%;[4] and 0.009% for microphthalmos in China from mass screening programs.[5] Data from a hospital cohort suggests patients with simple microphthalmos comprise between 0.05% and 0.11% ophthalmic patients.[6] There is great heterogeneity in the definition of nanophthalmos and microphthalmos which complicates interpretation of previous studies,[2,7–12] with a definition by axial length <21.00mm being the most inclusive.[10–12] Nanophthalmos / microphthalmos is associated with angle closure glaucoma;[1,13] and also with significant visual morbidity. In a recent series of nanophthalmic individuals from a Melanese population almost half had either unilateral or bilateral visual impairment.[11] There is a paucity of data for comparison. In view of this, we report data on the prevalence and characteristics of small eyes in British adults in the EPIC-Norfolk Eye Study, and review the definitions used for microphthalmos and nanophthalmos.

Method:

EPIC (European Prospective Investigation of Cancer) is a pan-European study that started in 1989 with the primary aim of investigating the relationship between diet and cancer risk.[14] The aims of the EPIC-Norfolk cohort were subsequently broadened to include additional endpoints and exposures such as lifestyle and other environmental factors.[15] The EPIC-Norfolk cohort was recruited in 1993-1997 and comprised 25,639 predominantly white European participants aged 40-79 years. The third health examination was carried out between 2006 and 2011 with the objective of investigating various physical, cognitive and ocular characteristics of participants then aged 48-91 years.[16] The third health examination was reviewed and approved by the East Norfolk

For peer review only

and Waverney NHS Research Governance Committee (2005EC07L) and the Norfolk Research Ethics Committee (05/Q0101/191) and was performed in accordance with the principles of the Declaration of Helsinki. All participants gave written, informed consent.

All EPIC-Norfolk Eye Study participants underwent a detailed health examination performed by trained nurses following standard operating procedures. Ocular biometry was measured by non-contact partial coherence interferometry using the Zeiss IOLMaster Optical Biometer (IOLMaster, Carl Zeiss Meditech Ltd, Welwyn Garden City, UK). Five measurements of both axial length (AL) and anterior chamber depth (ACD, defined as corneal epithelium to anterior crystalline lens surface) and 3 measurements of central keratometry were made to allow calculation of mean values. Refractive error was measured using an autorefractor (Model 500, Humphrey Instruments, San Leandro, California, USA). Three intraocular pressure (IOP) measurements were made for each participant using the non-contact Ocular Response Analyzer (ORA, Reichert Inc, Depew, NY) and the mean Goldmann correlated IOP (IOPg) calculated. Visual acuity was measured under standardized conditions at 4m using participants' normal method of distance vision correction and recorded on the LogMAR scale. Fundal photographs were taken of both eyes using a TRC-NW65 non-mydriatic retinal camera (Topcon Corporation, Tokyo, Japan) with Nikon D80 camera (Nikon Corporation, Tokyo, Japan). A masked, expert grader from the Moorfields Grading Centre measured vertical cup-disc ratio (VCDR). Systolic and diastolic blood pressures (BP) were taken from the right arm with the participant seated for 5 minutes. A stadiometer was used to record participant height to the nearest 0.1cm and weight was measured to the nearest 0.1 Kg using a body composition analyser (Tanita model TBF 300s, Chasmors Ltd, London, UK). Self reported data on education, occupation, alcohol intake and smoking status were recorded by questionnaire.

A small eye was defined by an axial length of <21.0mm in at least one eye in keeping with the broadest previously accepted definition for microphthalmos / nanophtahlamos[10–12] and being equivalent to 2SD below the population mean

For peer review only

value.[17] All investigations were performed on both eyes of each participant and the data from the eye with lower axial length used for analyses at the participant level, with the exception of visual impairment classification where data from both were used. Visual impairment was defined by the presenting vision in accordance with the International Classification of Diseases Update and Revision 2006[18] and the World Health Organization (WHO), which formally comprises categories 1 to 5 with categories 3 to 5 being blindness. To allow comparison with previous publications we defined blindness as a presenting visual acuity ≥1.3 logMAR in the better eye and low vision as a presenting visual acuity of >0.48 in the better eye (i.e. combination of moderate and severe visual impairment categories). Unilateral visual impairment was defined by using the eye with worse presenting visual acuity.

Statistical analysis was performed using SPSS version 20. Testing of normality was performed by the Kolmogorow-Smirnov method. Comparisons between participants with and without previous lens extraction were performed using the independent samples t-test or Mann-Whitney U test. Logistic regression was used to identify factors associated with presence of a small eye and Fisher's exact test to compare presence of visual impairment with the presence of a small eye.

Results.

Partial coherence interferometry data was available on 15,881 eyes of 8,033 participants, of which 4,442 participants were female (55.3%). Case numbers and overall prevalence values for small eyes stratified by axial length value are shown in table 1 and figure 1. Of the 8033 participants with axial length data, visual acuity measurements were available on 8016 (99.8%).

For peer review only

Table 1: Number of participants/ eyes and overall prevalence values (with 95% confidence intervals) by axial length (mm).

Axial length (mm)	a) Analysis by participant		b) Analysis by eyes	
	Number	Prevalence (95% CI)	Number	Prevalence (95% CI)
<21.00	96	1.195% (0.980 to 1.457)	132	0.831% (0.702 to 0.985)
<20.50	47	0.585% (0.441 to 0.777)	57	0.359% (0.277 to 0.465
<20.00	22	0.274% (0.182 to 0.414)	24	0.151% (0.102 to 0.225)
<19.00	14	0.174% (0.105 to 0.292)	14	0.088% (0.053 to 0.148)
<18.00	11	0.137% (0.077 to 0.245)	11	0.069% (0.039 to 0.124)
<17.00	4	0.050% (0.020 to 0.127)	4	0.025% (0.010 to 0.065)
<16.00	1	0.012% (0.003 to 0.069)	1	0.006% (0.002 to 0.035)
<15.00	1	0.012% (0.003 to 0.069)	1	0.006% (0.002 to 0.035)

Of the 96 participants, 20 were pseudophakic in both eyes, 6 were pseudophakic in one eye, 1 was aphakic in both eyes (congenital cataracts and nystagmus) and 1 aphakic in one eye and pseudophakic in the other. Defined by smallest eye, 26 participants had undergone previous lens extraction. Fourteen participants (15%) had a history of amblyopia or previous squint surgery. Seven participants (7%) had a history of previous laser iridotomy or surgical iridectomy. Table 2 shows the demographic and biometric characteristics of those with axial length <21.00mm.

For peer review only

Table 2: Demographic and biometric data presented as mean values with (standard deviation and range min: max value (range for all participants only), with [median values, IQR] shown for AL & ACD only. Comparisons with p values are between phakic and those with previous lens extraction.

	All, axial length <21.00mm	Phakic	Previous lens extraction	p value
Number	96	70	26	-
Age [years]	69.0 (8.8, 50.9 to 89.2)	66.3 (7.5)	76.5 (7.6)	<0.001
Sex	22M, 74F	13M/ 57F	9M/ 17F	0.11
AL [mm]	20.05 (1.26, 14.27 to 20.98) [20.53, 0.80]	20.45 (0.85) [20.61, 0.48]	18.96 (1.55) [18.91, 2.85]	<0.001
ACD [mm]	2.94 (0.69) [2.75, 0.78]	2.67 (0.44) [2.62, 0.44]	3.75 (0.71) [3.98, 0.92]	<0.001
Mean K [D]	45.24 (1.62, 41.71 to 51.19)	45.45 (1.65)	44.64 (1.41)	0.044
SE [D]	+3.63 (2.94, -5.50 to +8.38)	+5.04 (1.84)	-0.15 (1.71)	<0.001
Anisometropia, [D]	1.13 (1.23, 0.00 to 6.76)	1.20 (1.27)	0.94 (1.09)	0.37
V/A [logMAR]	0.31 (0.47, -0.20 to 1.68)	0.37 (0.53)	0.16 (0.24)	0.061
LogMAR difference between eyes	0.31 (0.44, 0.00 to 1.82)	0.38 (0.49)	0.12 (0.19)	0.012
IOP [mmHg]	15.7 (3.8)	15.6 (3.9)	16.0 (3.3)	0.63

Analysis of the difference in axial length between eyes showed a bimodal distribution (figure 2) with 19 participants (20%) comprising the second peak with a mean axial length difference of 5.63mm (SD 0.97) compared to 77 participants in the first peak with mean axial length difference of 0.45mm (SD 0.39).

Both univariable and multiple variable regression analyses investigating ocular biometric parameters in phakic eyes showed small eyes were associated with shallower anterior chamber depth, steeper corneal keratometry and higher spherical equivalent (all p<0.001, Table 3). Separate analyses were performed for other, non-ocular biometric parameters. For these, univariable logistic regression analyses showed female sex (OR 2.75, p<0.001), height (per 10cm, OR 0.46, p<0.001), weight (per 10Kg, OR 0.60,

For peer review only

p<0.001, body mass index (BMI, OR: 0.68, p=0.005) and systolic blood pressure (per 10mmHg, OR 1.11, P=0.029) were associated with the presence of a small eye. Multiple variable logistic regression models showed shorter height, lower BMI, higher systolic BP and lower IOP to be independent predictors of a small eye (Table 3).

Table 3: Univariable and multiple variable logistic regression analyses of factors associated with small eyes. Ref: reference category. For the multiple variable regression models (either a or b), only parameters reaching statistical significance in the respective univariable analysis were included, and only those in the final model shown.

For peer review only

a) Ocular biometric parameters: phakic participants			
Univariable regression	Odds ratio	95% CI	p value
Anterior chamber depth (per 1mm)	0.06	0.03 to 0.12	<0.001
Mean keratometry (per 1D)	2.16	1.82 to 2.57	<0.001
Spherical equivalent (per 1D)	2.67	2.35 to 3.03	<0.001
Multiple variable regression	Odds ratio	95% CI	p value
Anterior chamber depth (per 1mm)	0.02	0.01 to 0.08	<0.001
Mean keratometry (per 1D)	5.97	3.98 to 8.98	<0.001
Spherical equivalent (per 1D)	5.89	4.16 to 8.31	<0.001
b) Other parameters: all participants			
Univariable regression	Odds ratio	95% CI	p value
Age (per decade)	1.06	0.82 to 1.36	0.67
Female sex	2.75	1.70 to 4.43	<0.001
Height (per 10cm)	0.46	0.36 to 0.58	<0.001
Weight (per 10Kg)	0.60	0.51 to 0.72	<0.001
BMI (per 5 kg/m^2)	0.68	0.52 to 0.89	0.005
Social class			
Professional	Ref		
Managerial/technical	0.81	0.39 to 1.69	0.57
Skilled non-manual	0.91	0.40 to 2.09	0.82
Skilled manual	0.95	0.43 to 2.10	0.90
Partly-skilled	1.06	0.44 to 2.53	0.90
Unskilled	1.76	0.53 to 5.77	0.35
Education level			
Less than O level	Ref		
O Level	1.31	0.69 to 2.50	0.41
A level	0.94	0.57 to 1.55	0.81
Degree	0.93	0.50 to 1.76	0.83
Systolic blood pressure (per 10mmHg)	1.11	1.01 to 1.23	0.029
Diasolic blood pressure (per 10mmHg)	0.97	0.78 to 1.20	0.78
Self-reported alcohol intake			
No intake	Ref		
<7 units/wk	0.81	0.48 to 1.37	0.43
≥7 <14 units/wk	0.61	0.33 to 1.13	0.12
≥14 <21 units/wk	0.61	0.28 to 1.33	0.22
≥21 units/wk	0.70	0.38 to 1.28	0.25
Smoking status			
Never	Ref		
Ever	0.85	0.56 to 1.27	0.41
Intraocular pressure (mmHg)	0.95	0.90 to 1.01	0.09
Multiple variable regression	Odds ratio	95% CI	p value
Age (per decade)	0.89	0.68 to 1.17	0.40
Female sex	0.91	0.47 to 1.77	0.77
Height (per 10cm)	0.42	0.29 to 0.59	<0.001
BMI (per 5 kg/m^2)	0.69	0.53 to 0.90	0.006
Systolic blood pressure (per 10mmHg)	1.11	1.01 to 1.22	0.030
Intraocular pressure (mmHg)	0.93	0.88 to 0.99	0.030

For peer review only

Optic disc grading was possible on both eyes of 61/ 96 (64%) participants and at least one eye of 82/96 (85%) participants (right eyes: 12 missing, 9 un-gradable; left eyes: 14 missing, 14 un-gradable). Three participants (3/61, 4.9%) had vertical cup:disc ratio (VCDR) asymmetry of 0.2 or more, and 1 additional participant had an optic disc consistent with glaucoma (localized absence of neural rim, one eye only), giving an overall prevalence of 4/61 (6.6%, 95% CI: 2.6 to 15.7%) for glaucomatous optic neuropathy. No eye had a VCDR of ≥0.6. Five of 96 (5.2%) participants gave a diagnosis of “glaucoma” in their past medical history, of these only 1 had a diagnosis consistent with their optic disc photographs. Three participants had one optic disc with disc drusen. There were no cases of macular hypoplasia, macular schisis, coloboma or any other retinal abnormality associated with nanophthalmos.

Visual acuity data were available for all 96 participants and values are shown in table 2. One participant (1.0%) was classified as blind by the WHO definition (visual acuity of less than 1.3 logMAR) and 2/96 (2.1%) had any degree of visual impairment. Using a definition of visual impairment of >0.30 logMAR in the better eye to allow comparison with previous visual impairment studies, the prevalence was 5/96 (5.2%). The prevalence of blindness was significantly higher in EPIC-Norfolk participants with at least one eye of axial length <21.00mm compared to those without, whilst the overall prevalence of low vision was similar (Table 4). Unilateral visual impairment by all definitions was more common in EPIC-Norfolk participants with at least one small eye compared to those without (P≤0.001, Table 4).

For peer review only - http://bmjopen.bmj.com/site/about/guidelines.xhtml

Table 4: Percentages of bilateral and unilateral visual impairment in participants with one or both eyes with axial length <21.00mm (n=96) compared to all EPIC-Norfolk participants with no eye of axial length <21.00mm (n=7920) by Fishers exact test. Bilateral visual impairment is defined as both eyes with a visual acuity less than the respective value and unilateral visual impairment as one eye with a visual acuity less than the respective value.

logMAR	Snellen equivalent	Classification	Overall EPIC-Norfolk cohort (n=7920 total)		EPIC-Norfolk participants with small eyes (n=96 total)		p value
			n	Prevalence (95% CI)	n	Prevalence (95% CI)	
>1.30 better eye	<3/60; 20/400	WHO blindness	2	0.03% (0.00, 0.06)	1	1.0% (0.0, 3.1)	0.036
>0.48 better eye	<6/18; 20/60	Blindness and visual impairment; "low vision."	45	0.6% (0.4, 0.7)	2	2.1% (0.0, 5.0)	0.11
>0.22 better eye	<6/10; 20/32	UK driving standard	422	5.3% (4.8, 5.8)	7	7.29% (2.0, 12.56)	0.36
>0.30 better eye	<6/12; 20/40	Previous visual impairment studies, American driving standard	259	3.3% (2.9, 3.7)	5	5.2% (0.7, 9.7)	0.25
>1.0 worse eye	<6/60; 20/200	Unilateral visual impairment	120	1.5% (1.3, 1.8)	11	11.5% (5.0, 18.0)	<0.001
>0.48 worse eye	<6/18; 20/60	Unilateral visual impairment	470	5.9% (5.4, 6.5)	24	25.0% (16.2, 33.8)	<0.001
>0.30 worse eye	<6/12; 20.40	Unilateral visual impairment	1,341	16.9% (16.1, 17.8)	29	30.2% (20.9, 39.6)	0.001

Discussion:

There is minimal data describing the prevalence or characteristics of small eyes. In the EPIC-Norfolk Eye Study, the prevalence of a participant with an eye of axial length of <21.00mm was 1.20%, and 0.27% for those with an eye of axial length <20.00mm. Relative to existing data with estimated birth prevalences for microphthalmos being 0.002% to 0.017%;[4,5] and the prevalence of simple microphthalmos in hospital ophthalmic patients being between 0.05% and 0.11%;[6] small eyes are more common than previously reported.

Nanophthalmos is traditionally associated with a high prevalence of angle closure glaucoma[1,13] and both nanophthalmos and primary angle closure glaucoma have similar ocular phenotypes including a short axial length,

For peer review only - http://bmjopen.bmj.com/site/about/guidelines.xhtml

shallow anterior chamber, hyperopia, small radius of corneal curvature and a thick crystalline lens. In this study, the prevalence of glaucomatous optic neuropathy was estimated to be 6.6% in small eyes based on CDR, CDR asymmetry or rim abnormalities consistent with glaucoma. Othman and co-workers reported that 12/22 (55%) nanophthalmic individuals had occludable anterior chamber angles or glaucoma.[19] In the case series by Tay and co-workers of 17 individuals with nanophthalmos, no data on glaucoma prevalence is reported.[11] For comparison, the prevalence of glaucoma (open angle and closed angle combined) has been estimated to be 2.4% in European populations.[20] We did not calculate VCDR percentiles for the overall EPIC cohort as per the ISGEO (International Society Geographical & Epidemiological Ophthalmology) definition of glaucoma,[21] however no small eye in our series had a VCDR of ≥0.60. Crowston et al. reported optic disc size adjusted VCDR percentiles from the Blue Mountains Eye Study[22] and showed that for small discs (1.2mm vertical diameter) in non-glaucomatous eyes, the 97.5th percentile for VCDR was 0.60 (99th percentile: 0.62) whilst corresponding values for large optic discs (1.9mm diameter) were 0.75 and 0.83 respectively. Thus our prevalence of 6.6% should be considered as a minimum prevalence estimate for glaucoma in small eyes, and is likely to include those at highest risk for visual impairment over their lifetime.[21]

Review of recent studies reporting on nanophthalmos / microphthalmos shows great heterogeneity in the definitions used, with cases defined primarily by short axial length with for example, values of <21.0mm,[10–12] <20.9mm,[2] <20.5mm,[7] <20.0mm,[8] <18mm[2] or <17mm.[9] The original description of nanophthalmos (or pure microphthalmos) by Duke-Elder[1] is an eye "reduced in volume without the presence of other gross congenital abnormalities," "typical dimensions are 16-18.5mm sagittal," "hyperopia is the rule" and "the anterior chamber is typically shallow." The partial relaxation of the definition to its currently accepted form (of at least an axial length of <21.00mm) is likely due to the rarity of the condition. If an abnormally short eye is defined based on the lower 2SD and 3SD limits of mean population axial lengths, then the calculated limits are approximately 21.0mm and 20.0mm respectively.[16] In a previous study by our group investigating complications in small eyes

(<21.00mm) undergoing phacoemulsification and lens implantation, only axial length and the presence of abnormal IOP remained significant predicators of any complication in multiple variable regression analysis.[12] Complications were 15 times more likely in cases with axial length of <20.0mm (compared to those 20.00-21.00mm, p≤0.001). The differential complication rate supports the previous recommendation by Weiss et al.[2] that microphthalmos and nanophthalmos should be considered as 2 separate phenotypes, based on axial length. Based on the above it would appear reasonable to classify small eyes into microphthalmos (<21.0mm) and nanophthalmos (<20.0mm) respectively.

We found 1% of participants with small eyes were blind and 2% had low vision. When compared to all EPIC-Norfolk participants, blindness appears to be more common in those with a small eye (p=0.036, although case numbers were very low), but low vision was not (p=0.36). Unilateral visual impairment (defined by the worse seeing eye) was more common by all definitions (p≤ 0.001). When compared to data from population studies, the prevalence of visual impairment in EPIC-Norfolk participants is low overall, and values in those with small eyes is again low or similar. In the Blue Mountains Eye Study,[23] 4.6% had a visual acuity of 6/12 (20/40) or less in the better eye, and 14.4% had a visual acuity of 6/12 or less in their worse eye, whereas in our cohort of small eyes the equivalent percentages were 5.2% and 30.2%. In the Salisbury Eye Study, 9% of participants aged 75 to 84 years old had a visual acuity of <6/12 in their better eye;[24] whilst in the MRC study in Britain this value was 15% for those 75 to 84 years old.[25] There is minimal data on visual impairment in nanophthalmic individuals, with a recent study in a Melanesia population[11] (definition: axial length usually <21mm in at least one eye) reporting 5/17 (29%) had bilateral visual impairment and 9/17 (53%) had unilateral visual impairment (defined as <6/12 (20/40) Snellen in the better eye).

We found a marked bimodal distribution in axial length difference between eyes in individuals with small eyes, with 20% individuals having >3.5mm axial length asymmetry. A bimodal distribution in axial length difference has not

For peer review only

previously been described, with Weiss et al.[2] reporting a difference of only 0.4mm or less in a series of 21 patients with simple microphthalmos.

Our study has a number of limitations; these being primarily the absence of lens thickness and scleral thickness data to further characterize participants with small eyes. Additionally participants were not examined on a slit-lamp for eg. gonioscopy to determine the presence of an occludable anterior chamber angle (and therefore to determine if the glaucomatous optic neuropathy in our 5 cases were in the presence of an open or closed anterior chamber angle). Our prevalence value for glaucoma was based on glaucomatous optic neuropathy rather than glaucomatous optic neuropathy and visual field defect.[21] Comparisons of visual acuities were only performed in EPIC-Norfolk participants in whom axial lengths were measurable, and consequently this may have excluded those with visual impairment or blindness where axial length could not have been measured optically (ie. underestimating prevalence values). Additionally, those with visual impairment may have self-selected not to participate in the EPIC-Norfolk Eye Study, thus again underestimating case numbers.

In summary, the small eye phenotype was more common than previously reported, and our study provides prevalence values in British adults. There are no standardized definitions for microphthalmos or nanophthalmos; however based on current evidence, subdivision by axial length of <21.0mm for microphthalmos and <20.0mm for nanophthalmos appears reasonable. People with small eyes appear more likely to be blind or have unilateral visual impairment. The estimated prevalence of glaucomatous optic neuropathy in our cohort appeared to be lower than expected and warrants further investigation.

For peer review only

Figure legends:

Figure 1: Graph showing the prevalence of an axial length less than the value shown at the participant level (defined by eye with shortest axial length), and at the eye level.

Figure 2: Cumulative frequency distribution of axial length difference (asymmetry) between eyes for each participant. Note the bimodal distribution.

FINANCIAL SUPPORT:

Supported by Grant G0401527 from the Medical Research Council, UK and Grant 262 from Research into Ageing, UK. APK is funded by a Wellcome Trust Clinical Research Fellowship. PJF was also supported by the Richard Desmond Charitable Trust (via Fight for Sight, grant 1956). ACD, TP & PJF were supported by the National Institute for Health Research (NIHR) Biomedical Research Centre based at Moorfields Eye Hospital NHS Foundation Trust and UCL Institute of Ophthalmology. The views expressed are those of the author(s) and not necessarily those of the NHS, the NIHR or the Department of Health

COMPETING/ CONFLICTS OF INTEREST:

No conflicting relationship exists for any author.

COPYRIGHT STATEMENT:

The Corresponding Author has the right to grant on behalf of all authors and does grant on behalf of all authors, an exclusive licence (or non-exclusive for government employees) on a worldwide basis to the BMJ Group and co-owners or contracting owning societies (where published by the BMJ Group on their behalf), and its Licensees to permit this article (if accepted) to be published in BMJ Open and any other BMJ Group products and to exploit all subsidiary rights, as set out in our licence.

For peer review only

CONTRIBUTORSHIP

ACD performed the data analysis and drafted the manuscript. APK contributed to the data analysis. TP contributed to the data analysis. SH contributed to the conception and design of the study. RL contributed to the design of the study, and to data acquisition and management. DCB contributed to the conception and design of the study. KTK and PJF contributed to the conception and design of the study, and to data interpretation. All authors read and critically revised the manuscript. All authors approved the final manuscript.

DATA SHARING

The data sharing and preservation strategy in EPIC-Norfolk is in accordance with the Wellcome Trust data management and sharing policy. Full details about the study including contact information are on the website http://www.epic-norfolk.org.uk. Investigators wishing to work with EPIC data contact the EPIC management group through the website, letter, phone or fax, and proposals have to fulfil a number of criteria including that the work is within the bounds of consent given by participants and has been ethically reviewed and approved; there is no serious risk to the viability of continuing the cohort study for example, through offence to the participants from use of the data supplied; the science of the proposal has been satisfactorily peer reviewed and the proposal does not duplicate work already being done. Access to data for collaborators is provided through password protected website access. The large numbers of collaborators EPIC-Norfolk has locally, nationally and internationally (>300), as evidenced by collaborative publications, demonstrate the commitment to maximising the value of the study.

For peer review only

References:

1 Duke-Elder S. *System of Ophthalmology: Volume 3*. Henry Kimpton, London 1964.

2 Weiss AH, Kousseff BG, Ross EA, *et al.* Simple microphthalmos. *Arch Ophthalmol* 1989;**107**:1625–30.

3 Weiss AH, Kousseff BG, Ross EA, *et al.* Complex microphthalmos. *Arch Ophthalmol* 1989;**107**:1619–24.

4 Shah SP, Taylor AE, Sowden JC, *et al.* Anophthalmos, microphthalmos, and typical coloboma in the United Kingdom: a prospective study of incidence and risk. *Invest Ophthalmol Vis Sci* 2011;**52**:558–64.

5 Hu Z, Yu C, Li J, *et al.* A novel locus for congenital simple microphthalmia family mapping to 17p12-q12. *Invest Ophthalmol Vis Sci* 2011;**52**:3425–9.

6 Singh OS, Simmons RJ, Brockhurst RJ, *et al.* Nanophthalmos: a perspective on identification and therapy. *Ophthalmology* 1982;**89**:1006–12.

7 Steijns D, Bijlsma WR, Van der Lelij A. Cataract Surgery in Patients with Nanophthalmos. *Ophthalmology* 2013;**120**:266–70.

8 Yuzbasioglu E, Artunay O, Agachan A, *et al.* Phacoemulsification in patients with nanophthalmos. *Can J Ophthalmol* 2009;**44**:534–9.

9 Faucher A, Hasanee K, Rootman DS. Phacoemulsification and intraocular lens implantation in nanophthalmic eyes: report of a medium-size series. *J Cataract Refract Surg* 2002;**28**:837–42.

10 Wu W, Dawson DG, Sugar A, *et al.* Cataract surgery in patients with nanophthalmos: results and complications. *J Cataract Refract Surg* 2004;**30**:584–90.

11 Tay T, Smith JE, Berman Y, *et al.* Nanophthalmos in a Melanesian population. *Clin Experiment Ophthalmol* 2007;**35**:348–54.

12 Day AC, Maclaren RE, Bunce C, *et al.* Outcomes of phacoemulsification and intraocular lens implantation in microphthalmos and nanophthalmos. *J Cataract Refract Surg* 2013;**39**:87–96.

13 Kimbrough RL, Trempe CS, Brockhurst RJ, *et al.* Angle-closure glaucoma in nanophthalmos. *Am J Ophthalmol* 1979;**88**:572–9.

14 Riboli E. Nutrition and cancer: background and rationale of the European Prospective Investigation into Cancer and Nutrition (EPIC). *Ann Oncol* 1992;**3**:783–91.

For peer review only

15 Day N, Oakes S, Luben R, *et al.* EPIC-Norfolk: study design and characteristics of the cohort. European Prospective Investigation of Cancer. *Br J Cancer* 1999;**80 Suppl 1**:95–103.

16 Khawaja AP, Chan MPY, Hayat S, *et al.* The EPIC-Norfolk Eye Study: rationale, methods and a cross-sectional analysis of visual impairment in a population-based cohort. *BMJ Open* 2013;**3**. doi:10.1136/bmjopen-2013-002684

17 Foster PJ, Broadway DC, Hayat S, *et al.* Refractive error, axial length and anterior chamber depth of the eye in British adults: the EPIC-Norfolk Eye Study. *Br J Ophthalmol* 2010;**94**:827–30.

18 Pascolini D, Mariotti SP. Global estimates of visual impairment: 2010. *Br J Ophthalmol* 2012;**96**:614–8.

19 Othman MI, Sullivan SA, Skuta GL, *et al.* Autosomal dominant nanophthalmos (NNO1) with high hyperopia and angle-closure glaucoma maps to chromosome 11. *The American Journal of Human Genetics* 1998;**63**:1411–8.

20 Quigley HA, Broman AT. The number of people with glaucoma worldwide in 2010 and 2020. *Br J Ophthalmol* 2006;**90**:262–7.

21 Foster PJ, Buhrmann R, Quigley HA, *et al.* The definition and classification of glaucoma in prevalence surveys. *Br J Ophthalmol* 2002;**86**:238–42.

22 Crowston JG, Hopley CR, Healey PR, *et al.* The effect of optic disc diameter on vertical cup to disc ratio percentiles in a population based cohort: the Blue Mountains Eye Study. *Br J Ophthalmol* 2004;**88**:766–70.

23 Wang JJ, Foran S, Mitchell P. Age-specific prevalence and causes of bilateral and unilateral visual impairment in older Australians: the Blue Mountains Eye Study. *Clinical & Experimental Ophthalmology* 2000;**28**:268–73.

24 West SK, Munoz B, Rubin GS, *et al.* Function and visual impairment in a population-based study of older adults. The SEE project. Salisbury Eye Evaluation. *Investigative Ophthalmology & Visual Science* 1997;**38**:72–82.

25 Evans JR, Fletcher AE, Wormald RPL, *et al.* Prevalence of visual impairment in people aged 75 years and older in Britain: results from the MRC trial of assessment and management of older people in the community. *Br J Ophthalmol* 2002;**86**:795–800.

For peer review only

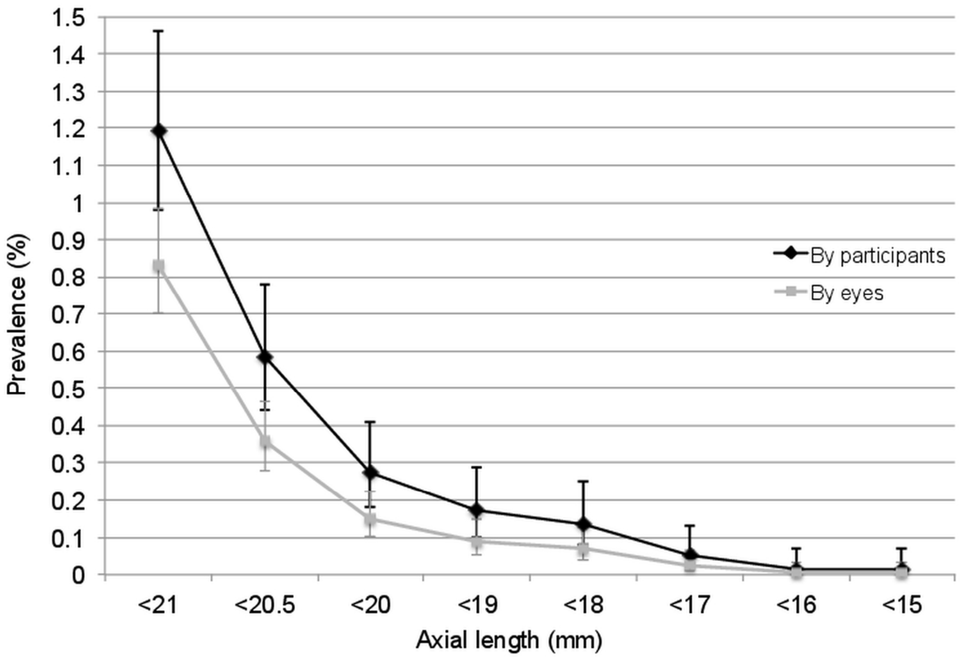

Figure 1: Graph showing the prevalence of an axial length less than the value shown at the participant level (defined by eye with shortest axial length), and at the eye level.
130x90mm (300 x 300 DPI)

view only

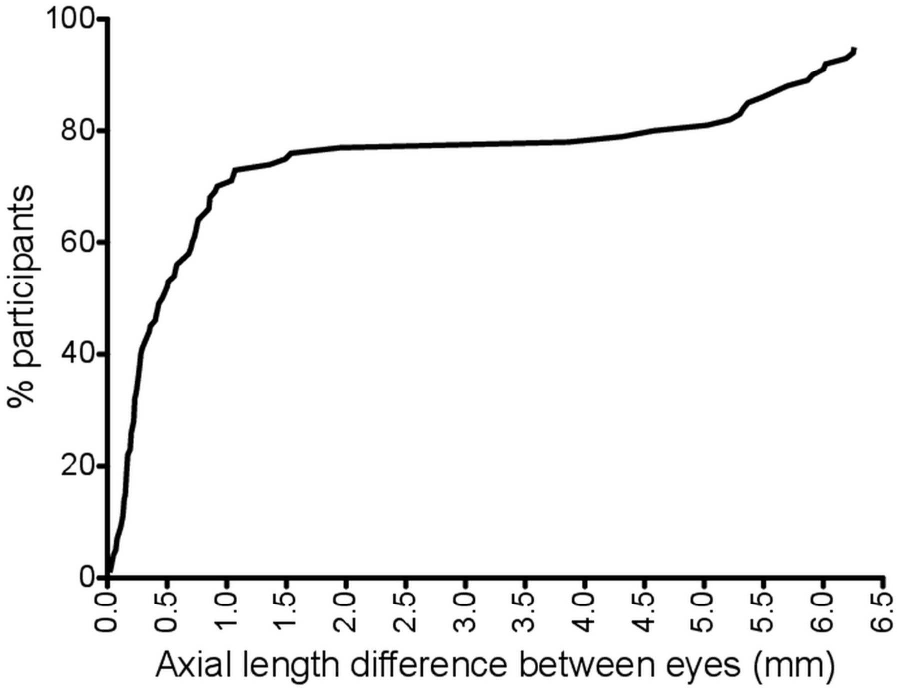

Figure 2: Cumulative frequency distribution of axial length difference (asymmetry) between eyes for each participant. Note the bimodal distribution.
120x90mm (300 x 300 DPI)
